# The Role of Phylogenetics in Discerning HIV-1 Mixing among Vulnerable Populations and Geographic Regions in Sub-Saharan Africa: A Systematic Review

**DOI:** 10.3390/v13061174

**Published:** 2021-06-19

**Authors:** George M. Nduva, Jamirah Nazziwa, Amin S. Hassan, Eduard J. Sanders, Joakim Esbjörnsson

**Affiliations:** 1 Department of Translational Medicine, Lund University, 205 02 Malmö, Sweden; george.makau_nduva@med.lu.se (G.M.N.); jamirah.nazziwa@med.lu.se (J.N.); ahassan@kemri-wellcome.org (A.S.H.); 2 Kenya Medical Research Institute (KEMRI)-Wellcome Trust Research Programme, Kilifi 80108, Kenya; ESanders@kemri-wellcome.org; 3 Centre for Tropical Medicine and Global Health, Nuffield Department of Medicine, The University of Oxford, Oxford OX1 2JD, UK

**Keywords:** HIV-1, phylogenetics, mixed epidemics, Sub-Saharan Africa, transmission dynamics

## Abstract

To reduce global HIV-1 incidence, there is a need to understand and disentangle HIV-1 transmission dynamics and to determine the geographic areas and populations that act as hubs or drivers of HIV-1 spread. In Sub-Saharan Africa (sSA), the region with the highest HIV-1 burden, information about such transmission dynamics is sparse. Phylogenetic inference is a powerful method for the study of HIV-1 transmission networks and source attribution. In this review, we assessed available phylogenetic data on mixing between HIV-1 hotspots (geographic areas and populations with high HIV-1 incidence and prevalence) and areas or populations with lower HIV-1 burden in sSA. We searched PubMed and identified and reviewed 64 studies on HIV-1 transmission dynamics within and between risk groups and geographic locations in sSA (published 1995–2021). We describe HIV-1 transmission from both a geographic and a risk group perspective in sSA. Finally, we discuss the challenges facing phylogenetic inference in mixed epidemics in sSA and offer our perspectives and potential solutions to the identified challenges.

## 1. Introduction

Molecular phylogenetic approaches have evolved into powerful tools in understanding pathogens and how they cause disease in human populations [1]. Based on genetic relatedness between pathogen strains, these studies have been coupled with epidemiological data to decipher transmission events in infected hosts [2]. This approach has several applications and has been used to understand the geographic distribution of a large number of pathogens (e.g., reconstructing the 2009 global spread of human influenza A H1N1 pandemic and, more recently, characterising the emergence and global spread of SARS-CoV-2) [3,4,5,6]. Phylogenetics and phylodynamics have also been used to reconstruct and date the emergence and early spread of HIV-1, to assess epidemic growth dynamics, to determine HIV-1 genetic diversity and the prevalence of antiretroviral resistance mutations, to infer putative transmission events, and to determine evolutionary rates and spread of specific HIV-1 strains [7,8,9,10,11,12,13,14,15]. In recent years, the phylogenetic analysis of HIV-1 sequences from individuals with known risk behaviour and/or geographic location has become a powerful tool to identify sources of infections that potentially could be targeted to reduce HIV-1 incidence [13,16,17,18,19,20,21,22]. However, most of these studies have been conducted in well-resourced countries with HIV-1 epidemics that are likely to differ in transmission dynamics compared with epidemics in Sub-Saharan Africa (sSA). For example, the HIV-1 epidemic in North America and Europe is concentrated amongst key populations—defined by UNAIDS as men who have sex with men (MSM), female and male sex workers (FSW/MSW), transgender people, people who inject drugs (PWID), and prisoners and other incarcerated people [23]. In contrast, the epidemic in sSA is mostly spread out among heterosexuals (HET, presumed heterosexuals) who have lower HIV-1 incidence and prevalence compared to key populations [24,25,26]. The term “general population” has been used where risk assessment data are not available for HET in sSA [27]. For ease in comparison between studies in this review, the term HET is used to refer to populations not belonging to either HIV-1 key populations or HIV-1 vulnerable populations (i.e., adolescent girls in sSA, orphans, street children, people with disabilities, and migrant or mobile workers as defined by UNAIDS (including miners, fishing communities, and long-distance truck drivers)) [23]. Additionally, the HIV-1 epidemic in sSA has revealed extensive geographic heterogeneity with HIV-1 hotspots (i.e., geographic areas and HIV-1 key populations with high HIV-1 incidence and prevalence) [25].

Targeting HIV-1 control strategies to HIV-1 hotspots has been proposed as a feasible approach to reduce the global HIV-1 incidence [28,29,30]. However, targeting likely needs to be guided by an in-depth understanding of the molecular epidemiology and drivers of local epidemics [31]. Existing reviews have summarised phylogenetic studies of transmission in concentrated epidemics and have offered perspectives on how transmission dynamics in the mixed epidemics of sSA could be assessed by phylogenetics [32]. In a review article from 1999, Dennis et al. reported high levels of both new and existing infections in high-risk populations in Kenya, South Africa, and Uganda [32]. In another review article from the African context, the PANGEA (Phylogenetics Additionally, Networks for Generalized Epidemics in Africa) consortium proposed to use phylogenetics to identify characteristics of individuals or groups most likely to be at risk of infection or at risk of infecting others [24]. However, to date, no published reviews have explicitly assessed the role of phylogenetics in discerning the mixing between geographies and populations in sSA. In this review, we provide an overview of the contribution of phylogenetic inference in dissecting HIV-1 mixing between geographic areas with varying HIV-1 prevalence, as well as HIV-1 mixing between key populations and HET in sSA.

## 2. Materials and Methods

### 2.1. Systematic Literature Review

#### 2.1.1. Information Sources

An exhaustive search of the PubMed database (https://pubmed.ncbi.nlm.nih.gov/ (accessed on 12 March 2021)) was carried out by analysing peer-reviewed research articles on HIV-1 phylodynamics in sSA published in English in 1995–2021. Review articles, book chapters, editorials, and articles published in other languages were excluded from the search.

#### 2.1.2. Search Strategy

First, we determined keywords and MeSH terms that could be used to identify research articles where phylogenetic approaches have been used to understand HIV-1 transmission in sSA. The MeSH terms (HIV-1) AND (Africa) were used to select HIV-1 articles from African countries. The keywords “phylogenetic analysis” OR “phylodynamics” OR “evolution” OR “phylogeny” OR “molecular epidemiology” OR “transmission” were used to widen the scope and to ensure that all relevant research articles were included. Filters on the year of publication, language, and article type were applied to refine the search.

#### 2.1.3. Selection Process

Two investigators carried out the selection process independently. The articles were manually screened, first by title, then by abstract, to assess relevance based on our eligibility criteria (i.e., description of HIV-1 mixing within and between geographic regions and risk groups). Any discordance between the two independent reviewers on the eligibility of articles was resolved through discussions for a consensus.

#### 2.1.4. Data Extraction and Data Analysis

Shortlisted articles were imported into EndNote X8 (Clarivate, Philadelphia, PA, USA) for further management and to compile the information presented in this review.

## 3. Results

**Figure 1:**
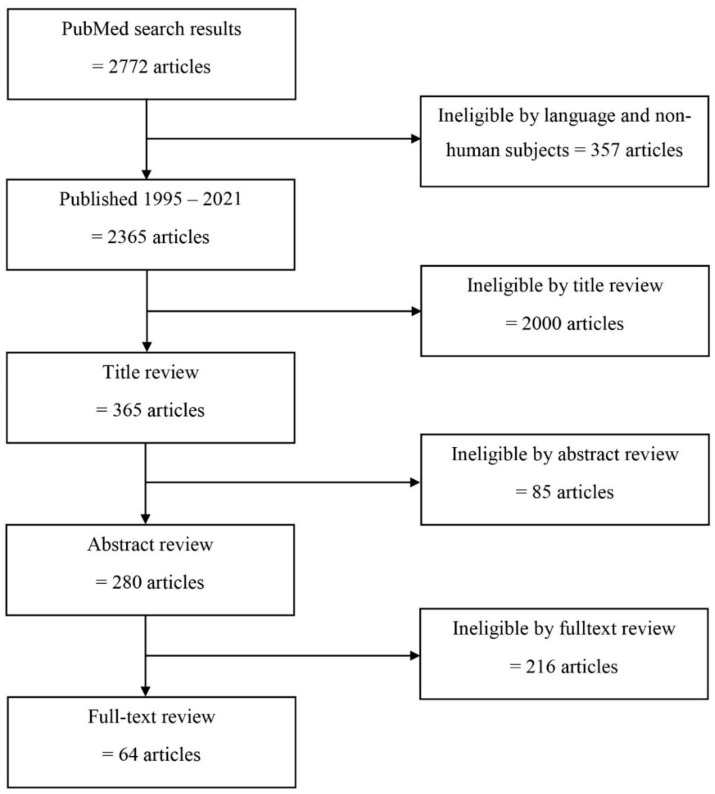
Study flowchart. Overview of the inclusion and exclusion of articles assessed in this review.

Based on a literature search done on 12 March 2021, 2722 articles were identified. Among these, 357 articles were not in English or involved nonhuman subjects, 2000 were ineligible by title review, 85 were ineligible by abstract review, and 216 were ineligible by a full-text review as they did not address HIV-1 transmission dynamics from a phylogenetic perspective (Figure 1). Sixty-four articles were considered eligible for full-text review, including 29 articles assessing geographic dispersion (Table 1) and 35 assessing HIV-1 mixing between HIV-1 populations in sSA.

### 3.1. HIV-1 Molecular Transmission Networks in sSA: What Has Been Done from a Geographic Perspective?

**Figure 2:**
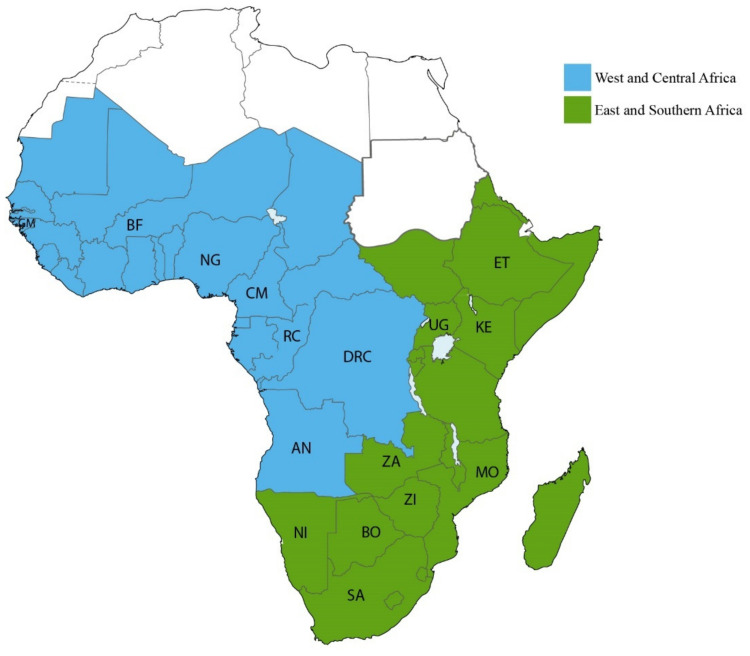
Subregions of Sub-Saharan Africa. A map showing different subregions of Sub-Saharan Africa as defined by UNAIDS. Countries belonging to Central and West Africa (*N* = 25) are coloured blue whereas countries belonging to Eastern and Southern Africa (*N* = 24) are coloured green. Where published information on HIV-1 transmission is available, the country code is included in the map. Countries belonging to Central and West Africa include Angola (AN), Benin, Burkina Faso, Cameroon (CM), Cape Verde, Chad, Central African Republic, Republic of the Congo (RC), Côte D’Ivoire, Democratic Republic of Congo (DRC), Equatorial Guinea, Gabon, The Gambia, Ghana, Guinea, Guinea-Bissau (GM), Liberia, Mali, Mauritania, Niger, Nigeria (NG), Saint Helena, Senegal, Sierra Leone, and Togo. Countries belonging to Eastern and Southern Africa include Burundi, Botswana (BO), Comoros, Djibouti, Ethiopia (ET), Eritrea, Kenya (KE), Lesotho, Madagascar, Malawi, Mauritius, Mozambique, Réunion, Namibia (NI), Rwanda, Seychelles, Somalia, Somaliland, Tanzania, South Africa (SA), Eswatini (former Swaziland), Uganda (UG), Zambia (ZA), and Zimbabwe (ZI).

The HIV-1 epidemic in sSA is driven by different HIV-1 subtypes, often geographically restricted [33,34]. Between 2010 and 2015, about 99% of the HIV-1 infections in Southern African countries and Ethiopia were subtype C, whereas the dominating HIV-1 strains in East Africa were sub-subtype A1 and subtype D [35]. The epidemic in West Africa is mainly driven by the circulating recombinant form (CRF) 02_AG, sub-subtype A3, and subtype G [12,35,36]. In contrast, the HIV-1 epidemic in Central Africa is more complex and diverse, and most HIV-1 subtypes have been found in this region [35,37]. In this review, we grouped the assessment of HIV-1 transmissions in sSA into two geographic regions according to the UNAIDS classification (https://aidsinfo.unaids.org/ (accessed on 20 January 2021)): East and Southern Africa; and West and Central Africa (Figure 2).

#### 3.1.1. HIV-1 Transmission in West and Central African Countries

Nigeria is the most populous country in sSA. A recent study by Nazziwa et al. used 1442 *pol* sequences (collected in 1999–2013) from four geopolitical zones in Nigeria (Southwest, North Central, Northeast, and Northwest) to reconstruct HIV-1 transmission dynamics [12]. Phylogeographic analyses suggested that HIV-1 first emerged and expanded within the large urban centres (Lagos and Abuja), before migrating to smaller and more rural areas. Abuja, the capital city of Nigeria, was estimated to be the geographical origin of both subtype G and CRF02_AG in Nigeria. In addition, the analysis indicated that one single introduction resulted in the main Nigerian subtype G epidemic (time to the most recent common ancestor, tMRCA; 1987). In contrast, the CRF02_AG had multiple introductions which expanded into larger subepidemics (tMRCAs; 1974, 1972 and 1961) [12]. Another study in Guinea-Bissau by Esbjörnsson et al. (based on 82 Guinean HIV-1 *env* sequences collected 1993–2008) found that the dominating HIV-1 strains were CRF02_AG and sub-subtype A3. In line with the study by Nazziwa et al., both subepidemics originated in the capital before dispersing out to smaller and more rural areas [36]. Interestingly, although the two HIV-1 strains were introduced into the country around the same time (median estimates 1976–1981), the phylogeographic analysis suggested that the CRF02_AG strain started to migrate to more rural areas almost instantly after being introduced into the Capital Bissau. In contrast, sub-subtype A3 was estimated to have circulated within the capital for approximately 10 years before migrating to more rural areas of the country. The underlying reasons for this, however, remain to be determined.

In Cameroon, Véras et al. used 291 HIV-1 CRF02_AG *pol* sequences collected in 1996–2007 with geographic information system (GIS) data to assess HIV-1 transmission dynamics. Seventy percent of the sequences were found in three distinct clusters, suggesting several subepidemics with different origins [38]. Southern Cameroon has denser human mobility networks compared to the rest of the country; a large cluster comprising sequences from southern Cameroon was identified, suggesting that human mobility may play a role in increasing HIV-1 transmission. In another Cameroonian study, based on 336 HIV-1 *gag*, *pol*, and *env* sequences collected in 1996–2004, two HIV-1 CRF02_AG Cameroonian clusters were identified [39]. Interestingly, both clusters were estimated to have originated in Yaoundé, the capital of Cameroon, before spreading to the Littoral and West regions of Cameroon and remote areas in the South and East. In a study in the Democratic Republic of the Congo (DRC), Faria et al. used a phylogeographic approach to analyse 346 HIV-1 *pol* sequences (subtypes A1, C and D) collected in 2008 from four locations—the capital Kinshasa, Matadi (West DRC), Mbuji-Mayi (Central DRC), and Lubumbashi (South DRC) [9]. Mbuji-Mayi was suggested as the origin of the subtype C epidemic, whereas the origin for subtypes A1 and D was Kinshasa. The study also indicated that several group M HIV-1 strains had spread from the DRC to other countries. In another study, analysis of *env* C2V3 sequences collected at multiple sites in the DRC (from Bwamanda in North, Kisangani and Mbuji-Mayi in Central, the Capital Kinshasa, Lubumbashi and Likasi in South), and the Republic of the Congo (from the Capital city Brazzaville, and Porte-Noire in West) suggested that HIV-1 dispersed from Kinshasa to Brazzaville, as well as from Bwamanda and Kisangani [8,40]. The authors suggested that good transport connectivity and human mobility linked to mining activities may have been involved in the rapid expansion of HIV-1 spread between Kinshasa, Brazzaville, Lubumbashi, and Mbuji-Mayi [8].

The HIV-1 epidemic in Angola is one of the most diverse in sSA, and all HIV-1 group M subtypes and several CRFs have been identified here [37,41,42]. In a study by Bártolo et al., 364 HIV-1 *pol* sequences collected in 1993–2010 from Luanda and seven other provinces in Angola were analysed. The results indicated that 36% of the sequences formed relatively small Angolan clusters. Seventy-four percent of the sequences in the identified clusters were from Luanda, indicating extensive local transmission and much lower transmission (24% of the clusters) beyond the capital city of Luanda [42].

#### 3.1.2. HIV-1 Transmission in East and Southern Africa Countries

The HIV-1 epidemic in Kenya is diverse and has had multiple and separate introductions [10,43,44]. Hue and colleagues performed a phylogeographic analysis based on 153 sequences collected in Kilifi county in 2008–2009 together with published Kenyan sequences to investigate how HIV-1 transmission in rural Coastal Kenya related to the region [43]. It was observed that 73% of the HIV-1 sub-subtype A1 sequences from Kilifi clustered with sequences from other areas in Kenya and that there was substantial clustering with strains from other East African countries, such as Uganda and Tanzania, possibly indicating HIV-1 transmission links between these countries. HIV-1 transmission in Uganda has been well studied, especially in rural Southwestern Uganda (which is suggested to be the geographic origin of HIV-1 sub-subtype A1 and subtype D in Uganda) [45]. To understand HIV-1 transmission dynamics in Uganda, Ssemwanga et al. used 3796 HIV-1 *pol* sequences collected between 2003 and 2015 from Southwestern, Central, and Eastern Uganda [46]. HIV-1 subtype A infections were more common in Central Uganda, whereas subtype D infections were more common in Southwestern Uganda. The study also found a high proportion of localized clustering among sequences from Southwestern Uganda and significant virus export from this region to other regions. However, no virus introductions into this region were observed. In another study, Yebra et al. used 162 HIV-1 *pol* sequences collected in 2005–2010 from Kampala, Masaka, and Entebbe and 414 previously published *pol* sequences from Rakai, Kampala, and Entebbe and observed that HIV-1 subtype D initially spread from the rural Southwest, then to the Capital Kampala, before spreading to areas around Lake Victoria [45]. In Ethiopia, the HIV-1 epidemic is dominated by two phylogenetically distinct subtype C types—the Ethiopian HIV-1 C’-ET, and the East African HIV-1 C-EA [47]. Arimide et al. used 301 HIV-1 *pol* sequences collected in 2003–2013 from Gondar (Northern Ethiopia) to define and understand the transmission dynamics among these variants. The study showed that the C-EA sequences in Gondar clustered with sequences from other East African countries and that multiple introductions of the South African subtype C (C-SA) were observed in Gondar [47].

In Southern Africa, the mass migration of people into South Africa from neighbouring countries has been suggested to have an impact on the local HIV-1 epidemic [48,49]. To understand the transmission dynamics of HIV-1 within South Africa and its neighbouring countries, Wilkinson and colleagues analysed 15257 HIV-1 subtype C southern African sequences [50]. The analysis indicated that Johannesburg and KwaZulu-Natal were the main epicentres of HIV-1 dissemination in South Africa. Viruses from KwaZulu-Natal spread to the Northern regions close to the Mozambican and Swaziland borders, and to Johannesburg, whereas viruses from Johannesburg spread to KwaZulu-Natal, Kimberly, Bloemfontein, Mpumalanga, and Western and Eastern Cape. Another study quantified the contribution of local transmission and external introductions to the HIV-1 incidence specifically in KwaZulu-Natal [51]. Phylodynamic analysis of 1068 HIV-1 *pol* sequences collected in 2011–2014 in KwaZulu-Natal together with 11,289 subtype C sequences from Southern African countries revealed multiple HIV-1 introductions into KwaZulu-Natal from other locations in South Africa and neighbouring countries. The majority of the virus introductions in this study occurred in the early stages of the South African HIV-1 epidemic during the 1990s, where human movements played a role in driving the epidemic and sustaining high HIV-1 incidence in KwaZulu-Natal. In addition, 35% of new infections in KwaZulu-Natal were due to HIV-1 imports from other regions. To understand the structure of the local HIV-1 epidemic in periurban Botswana, Novitsky et al. analysed 2219 HIV-1 *env* sequences (785 sequences from Mochudi, 190 sequences from other locations in Botswana, and 1244 non-Botswana sequences) [52]. Close clustering of sequences originating from Mochudi suggested that the HIV-1 epidemic in Mochudi was dominated by locally circulating HIV-1 variants. Moreover, none of the Mochudi sequences clustered with non-Botswana sequences.

#### 3.1.3. HIV-1 Transmission beyond Borders

Few studies have investigated the geographic mixing of HIV-1 between different African regions.

To shed light on the dissemination of HIV-1 CRF02_AG in Central and West Africa, Yebra et al. applied phylodynamic analysis to 1247 HIV-1 *env* and 1478 HIV-1 *pol* sequences collected 1984–2013 from 19 African countries. The analysis indicated that CRF02_AG originated from Cameroon from where it spread to other Central and West African countries [53]. To further characterise the CRF02_AG epidemic in West and Central Africa, Mir et al. used 2246 HIV-1 *pol* sequences collected 1990–2013 from 20 African countries [54]. The study indicated that the current CRF02_AG diversity resulted from the spread of a small number of founder strains from Central to West Africa in the period of 1960–1980. The study identified five different CRF02_AG variants, four of which were restricted to Cameroon and one that grew out into West Africa. In addition, other phylogeographic studies have indicated Cameroon as the epicentre of the dissemination of HIV-1 CRF11_cpx to Central African Republic, Chad, Gabon, and Equatorial Guinea. However, it has also been suggested that CRF06_cpx spread from Burkina Faso to Mali, Nigeria, and the rest of West-Central Africa [55,56].

A phylogeographic study of the dissemination routes of HIV-1 subtype G in West and Central Africa by Delatorre et al., using 305 HIV-1 *pol* sequences collected in 1992–2011 from 11 countries, showed that the African subtype G epidemic could be divided into two subepidemics according to sequence location, i.e., West and Central Africa [57]. Sequences from West Africa were further subdivided into two large monophyletic clusters that were nested within the Central African variant. One of the Western African variants emerged from Nigeria and spread to Benin, Cameroon, Equatorial Guinea, Ghana, and Senegal. The other West African variant emerged from Togo and/or Ghana from which it spread to Nigeria and then to Benin, Cameroon, Gabon, and Senegal [57].

To reconstruct the HIV-1 transmission dynamics of subtype C in East Africa, Delatorre et al. analysed 1981 *pol* sequences collected in 1990–2010 from 13 countries in Central, East, and Southern Africa [58]. Subtype C sequences from East Africa (Burundi, Ethiopia, Kenya, Tanzania, and Uganda) formed one large monophyletic cluster separate from sequences from Southern Africa. In addition to the East African C variant, another monophyletic cluster exclusive to Ethiopia was observed. The East Africa subtype C cluster disseminated from Burundi and later spread to other East African countries where local epidemics were established [58]. A later study including sequences collected in recent years (2013–2016) showed that most of the East African subtype C sequences still clustered into one monophyletic variant, consistent with strong interconnectivity between population centres across the East African region, which has likely fostered the rapid growth of the HIV-1 subtype A1, C, and D epidemic [59,60].

A comparative genetic analysis of HIV-1 subtypes A1, C, and D using 8701 *pol* sequences collected in 1996–2011 from DRC, Burundi, Kenya, Rwanda, Tanzania, and Uganda by Faria et al. indicated that subtypes A1 and D originated from DRC and that sequences from the same regions clustered closely together [9]. Additionally, 80% of total transmissions occurred within national borders and only 20% of transmissions were due to cross-border virus movements. Furthermore, Rwanda, DRC, and Tanzania were identified as the main exporters of subtype C in the Central and Eastern Africa region, whereas Uganda was the source of subtypes A1 and D.

To understand how human migration has influenced HIV-1 diversity and spread in Southern Africa, Wilkinson et al. performed a phylogeographic analysis of 11,289 sequences collected from DRC, Tanzania, Zambia, Malawi, Mozambique, Zimbabwe, Botswana, Namibia, Swaziland, Lesotho, and South Africa. The study showed that the high level of subtype C diversity in South Africa was linked to multiple HIV-1 introductions into the country [49]. Zambia, Botswana, Malawi, and Zimbabwe contributed to most of the HIV-1 introductions into South Africa between 1985 and 2000. However, South Africa also contributed to HIV-1 export to its neighbouring countries. HIV-1 mixing between Zimbabwe and other neighbouring countries (South Africa, Botswana, Zambia, Malawi, Mozambique, and Tanzania) has also been reported in a study by Dalai et al. [61]. Moreover, subtype C sequences from Southern and Central Africa have been shown to cluster closely together but separate from other subtype C sequences from other parts of the world, suggesting strong HIV-1 panmixia in Southern Africa [48,62].

#### 3.1.4. Conclusion Phylogeographic Linkages in sSA

In summary, the HIV-1 epidemics in West and Central Africa seem to have emerged and expanded within urban areas before spreading to rural areas—possibly driven by human mobility [12,36,39,42]. In other instances, HIV-1 mixing between rural and urban locations, as well as across national borders, has also been observed [9,42,43,47]. Some HIV-1 subepidemics appear to be localized in specific communities where HIV-1 mixing with neighbouring communities is not observed [54]. In contrast, in other settings localized HIV-1 subepidemics serve as important sources of HIV-1 infection to neighbouring communities [47,48]. Furthermore, human migration linked to economic activities such as mining and fishing may contribute to increased HIV-1 transmission [9,49,63].

### 3.2. The Role of HIV-1 Key and Vulnerable Populations in Mixed HIV-1 Epidemics: A Risk Groups Perspective

**Figure 3:**
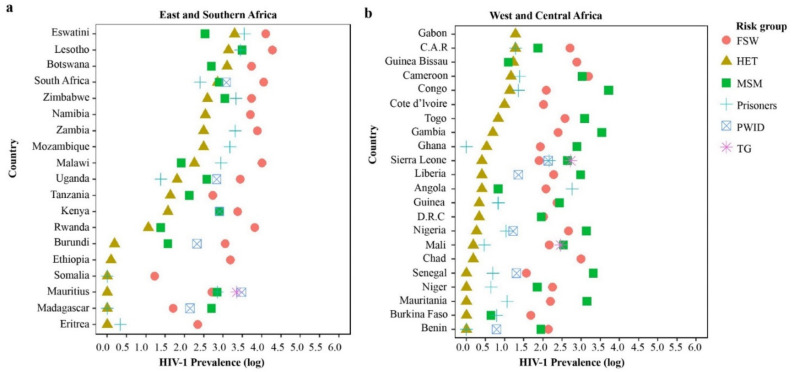
HIV-1 prevalence in different risk groups in sub-Saharan Africa (sSA). A comparison of national estimates of HIV-1 prevalence in the heterosexuals (HET) and among vulnerable populations in sSA as reported by UNAIDS in 2020 (https://aidsinfo.unaids.org/ (accessed on 20 January 2021)). East and Southern African (**a**), and West and Central African (**b**) regions were grouped together, respectively. The countries in each region were arranged in increasing HIV-1 prevalence among (HET), and HIV-1 prevalence data have been transformed into a log scale on the x-axis. Different risk groups are coloured as shown in the legend (Red: female sex workers; Brown: HET; Green: men having sex with men; Sky Blue: prisoners; Dark Blue: PWID; and Pink: transgender persons).

The early HIV-1 epidemic in sSA was exclusively defined as heterosexual and involving FSW and long-distance truck drivers [64,65,66]. The role of other HIV-1 key populations such as MSM and PWID was not apparent and this, coupled with ethical-legal hurdles, led to the exclusion of these key populations from early HIV-1 responses in sSA [67,68,69]. HIV-1 key populations in sSA are strongly affected by legal and social stigma, where risk behaviour associated with these populations (e.g., same-sex behaviour) are often criminalized [70]. As a consequence, individuals in these populations often withhold risk information, which results in limited HIV-1 research involving key populations [27]. Additionally, there is evidence of overlapping sexual networks and phylogenetic linkages between HIV-1 key populations and HET, which may have implications for the dynamics of HIV-1 spread [44,65]. Figure 3 summarises HIV-1 prevalence estimates among HIV-1 key and vulnerable populations relative to HET in different regions of sSA. In general, HIV-1 key populations have higher HIV-1 prevalence compared to HET in all sSA countries (with the exception of MSM in Eswatini, Malawi, Botswana, and Guinea Bissau).

#### 3.2.1. HIV-1 Phylogenetic Linkages Involving Heterosexual Transmission

Data on HIV-1 phylogenetic linkages involving HIV-1 key and vulnerable populations are not available in most sSA countries. Consequently, and albeit largely under sampled, most studies investigating HIV-1 networks have focused on HET transmission. In Botswana, a phylogenetic analysis of 1247 HIV-1 subtype C *env* sequences (collected in 2010–2013) by Novitsky et al. found 233 clusters, the majority of which were HET transmission pairs, and where the largest cluster involved 18 individuals [71]. This study was conducted in Mochudi, a periurban community, and it proposed tracking HIV-1 transmission clusters at the community level and extinguishing them, one by one, through targeted interventions. In a similar setting in South Africa, another analysis by Sivay et al. reported partial transmission chains constructed among young women attending high school in rural South Africa (in the context of missed sampling among males in the community) and revealed a stable local epidemic with no evidence of super-spreading events or large networks [72]. In addition, this study also showed that recent HIV-1 transmissions may play a key role in driving the local HIV-1 epidemic. In Zambia, a phylogenetic analysis of 149 married couples by Trask et al. showed that the majority (87%) of couples were epidemiologically linked [73]. However, 13% of pairs in the study had distantly related viruses, suggesting possible extramarital HIV-1 transmission. In Rwanda, a phylogenetic analysis of men and high-risk women in the context of multiple heterosexual partnerships by Rusine et al. identified only three potentially linked transmission pairs [74]. In the DRC, a study by Rubio-Garrido et al. using 165 newly generated HIV-1 *pol* sequences representing adults and majority paediatric individuals found only four clusters, one of which had sequences from children with no epidemiological links, indicating under sampling in otherwise denser networks [75]. Small clusters have also been observed in Ethiopia by Arimide et al., where data on MSM and PWID is not available, but the epidemic among FSW and HET is acknowledged [47]. Overall, multiple studies in all geographic regions of Sub-Saharan Africa have characterised HIV-1 phylogenetic linkages involving heterosexual transmission. The majority of these studies identified only small clusters, highlighting challenges in sampling coverage which results in many missing links in otherwise large networks [76].

#### 3.2.2. HIV-1 Phylogenetic Linkages among MSM

In the context of homosexual transmission, phylogenetic studies in East Africa have demonstrated extensive clustering among MSM [10,44,77,78]. Studies in Coastal Kenya, have demonstrated extensive clustering of HIV-1 *pol* sequences from men who have sex with men only (MSM only) and bisexual men, suggesting that bisexual MSM may link infections across different risk groups although such linkages may only be modest as observed in Coastal Kenya [10,44]. In West Africa, studies in Nigeria have observed clustering among MSM in a cohort involving a majority (62%) bisexual men [79,80]. Relatively large clusters (with up to 15 individuals per cluster) have been found in this cohort. Interestingly, 37% of bisexual men in this cohort were in clusters involving MSM [80]. In this cohort, clustering between newly infected MSM and previously diagnosed MSM has been reported, indicating ongoing transmission among MSM, the majority of whom were not in treatment and did not report consistent condom use. Elsewhere in the region, phylogenetic clustering analysis of 67 Senegalese MSM (of whom 80% reported to be married) identified 15 transmission clusters, three of which involved MSM from multiple regions in Senegal, indicating linked MSM networks with a wide geographic presence [81]. High numbers of MSM having female sex contacts and exclusive clustering among Senegalese MSM have also been reported by Ndiaye and colleagues [82]. Although cross-risk groups linkages between MSM and HET were not reported in either of these studies in West Africa, such mixing could be expected, to some extent, as has been observed in East Africa [44]. Overall, although MSM in the majority of cohorts in sSA often report being married or having female sex partners, phylogenetic evidence of HIV-1 transmission often reveals MSM exclusive clusters and only a few clusters involving HIV-1 sequences from MSM and HET, suggesting limited mixing.

#### 3.2.3. HIV-1 Phylogenetic Linkages among PWID

Phylogenetic studies involving PWID in sSA are exceedingly rare and have only been reported at a subnational scale in Kenya [44,78]. Nduva et al. used 658 sequences to investigate HIV-1 phylogenetic linkages involving MSM, FSW, PWID, and HET in Coastal Kenya [44]. Whereas MSM, FSW, and HET were found in several small clusters (indicating introduction from multiple sources), the vast majority of PWID sequences were found in one large PWID-exclusive cluster suggesting introduction from one single source and long-term gradual spread within the PWID in Coastal Kenya. Phylodynamic analysis of PWID sequences in this study suggested that HIV-1 infections had increased steadily among PWID since the date of origin in 1987. Additionally, unlike in previous studies (non-African) where PWID sequences clustered with exceptionally low genetic diversity, the genetic diversity among PWID in the Coastal Kenyan cluster was high [17,21,83]. The reason for this could be long times between infection and sampling dates and/or low sampling density among PWID in the region. Overall, studies so far suggest separate transmission for PWID with limited overlap between other key populations. However, more research is warranted as the molecular epidemiology of PWID in sSA is largely understudied.

### 3.3. Phylogenetic Analysis to Examine HIV-1 Mixing between Risk Groups

Very few studies in sSA have investigated HIV-1 linkages involving individuals belonging to different risk groups. In Southern Africa, Bártolo et al. assessed HIV-1 phylogenetic linkages in Angola using 364 HIV-1 *pol* sequences collected in 1993–2010 and identified 48 transmission clusters (size range: two to seven) [42]. More than half of the clustering sequences did not have risk group information. However, three clusters involving mixing between MSM and females were identified, suggesting HIV-1 genetic mixing between HET and MSM. In South Africa, Wilkinson and colleagues detected phylogenetic mixing between HET and MSM, where linkages involving two MSM (infected through homosexual contact) and an incarcerated man (infected in a prison setting) were found within a large cluster dominated by HET (including female individuals) [84]. HIV-1 mixing involving bisexual MSM and HET has also been reported in Cape Town, South Africa [85].

In West Africa, phylogenetic intermixing of HIV-1 variants between HET women and MSM has also been documented in Senegal, where sequences from HET females were found among MSM clusters [86]. Another study has reported on the intermixing of HIV-1 between MSM and HET in Togo [87]. The authors describe extensive clustering among 79 MSM, where at least 40% of MSM were found in recent transmission chains of two to seven sequences, and where almost half (49%) of MSM were found in one major CRF02_AG cluster, indicating infections within a close network. Additionally, in this study, a comparison of 950 published HIV-1 sequences from HET, perinatally infected infants, and MSM indicated HIV-1 mixing between MSM and HET because strains from infants and HET females were found among MSM-dominated clusters.

In East Africa, two studies in Kenya have reported limited mixing between key populations and HET [44,77]. Bezemer et al. found only one single transmission pair of an MSM and a known HET female partner in Coastal Kenya—indicating infrequent HIV-1 mixing between MSM and HET in Coastal Kenya [77]. A follow-up study by Nduva et al. used a larger sample size to study mixing between MSM, PWID, FSW, and HET in Coastal Kenya and found that only 7% of the clusters had MSM and HET sequences, indicating limited mixing between MSM and HET in Coastal Kenya [44].

In Uganda, phylogenetic clustering has been studied among Lake Victoria’s fishing communities (considered an HIV-1 vulnerable population) [88,89,90], and HIV-1 mixing between fishing communities and HET residing in in-land regions has been reported [90,91]. Grabowski et al. showed that HIV-1 diversity is similar both within and between fishing communities and with HET in surrounding communities [91]. In a different study, phylodynamic analysis of sequences from FSW, fishing communities, and HET identified only a few small clusters of exclusively HET individuals [45]. Although the sample size and the sampling coverage were low, no mixing between risk groups was observed. However, in the context of missed sampling of sex partners of FSW, a study in Kampala observed clustering among FSW, suggesting infection from the same source—possibly linked to frequent partner exchange among FSW [92]. Overall, multiple studies have provided evidence of HIV-1 phylogenetic linkages between HIV-1 key populations and HET in Sub-Saharan Africa. A common observation in most of these studies is clustering between HET females and MSM, in addition to the expected links between HET and FSW owing to sex work. HIV-1 mixing appears to be at relatively low rates across the region (although this has been difficult to quantify empirically because of the dearth of HIV-1 sequence data from MSM, FSW, and PWID).

### 3.4. Phylogenetic Analysis to Examine Sources and Direction of HIV-1 Transmission between HIV-1 Key and Vulnerable Populations and HET in sSA

Few studies have investigated the directionality in HIV-1 transmission involving different risk groups in sSA. Recent phylogenetic analyses have shown that fishing communities do not serve as a source of HIV-1 infection to much larger populations with lower HIV-1 prevalence in Uganda [46,90,93]. In Senegal, Nascimento et al. showed that 3.2% of infections in HET females were acquired from MSM, whereas 0.3% infections among MSM were acquired from HET females [94]. In Nigeria, a phylodynamic analysis of HIV-1 *pol* sequences from MSM and HET females by Volz et al. estimated a 9.1% virus flow from MSM to HET females and 0.2% HIV-1 transmissions from HET females to MSM [95].

Dennis et al. evaluated HIV-1 phylogenetic and behavioural characteristics among 45 newly diagnosed and acutely infected HIV-1 individuals (index partners) and their referred HET partners in Malawi [96]. None of the 45 index partners were closely linked phylogenetically. However, most index partners were linked with their chronically infected HET partners, highlighting the contribution of chronic infections to new HIV-1 transmissions. Another phylogenetic study by Jennes et al. analysed 46 HIV-1 concordant positive HET couples in Dakar, Senegal, to understand the dynamics and risk factors of within-couple HIV-1 transmissions [97]. The analysis showed that male partners were the most likely index partners (and hence the source of infection) to married women.

Phylogenetic studies have also revealed the role of age-disparate HET relationships in perpetuating local HIV-1 transmission in Sub-Saharan Africa [46,98,99]. In Uganda, Ssemwanga et al. found that HET individuals older than 25 years were more likely to appear in phylogenetic clusters than younger individuals [48]. This study suggested that high-risk HET behaviour involving older individuals living with HIV-1 may drive recurring new infections. In Botswana, a country-wide study involving 6078 sequences by Novitsky et al. identified 984 phylogenetically distinct clusters, revealing complex HIV-1 phylogenetic linkages with mixing between different communities and geographic regions [99]. This study suggested that HIV-1 may first be transmitted from older women to middle aged men, followed by transmission from these men to young women. This HIV-1 transmission cycle had been described earlier in KwaZulu-Natal, South Africa, where HIV-1 is first transmitted from women aged 25–40 years to men aged 25–40 years who would then transmit to girls and young women (15–25 years) [98]. Overall, research has shown that key populations may contribute a modest fraction of infections to the HET population and that key populations may be a sink and not the major source of infections in the mixed epidemic. Further research is needed to reveal the drivers of the HIV-1 epidemic in sSA [90,100].

## 4. Perspectives, Challenges, and Potential Solutions with Phylogenetic Inference in sSA

First, most sequence-based studies in sSA have focused on transmitted drug resistance, and more phylogenetic studies dissecting how HIV-1 in different populations mix and spread are warranted. Second, there is a need to incorporate mobility networks into the phylogenetic spatiotemporal models to quantify the movement patterns and links between urban and rural communities more precisely. Although these mobility methodologies have been developed and used to quantify the impact of human mobility on malaria transmission in different African countries, including Kenya and Madagascar, their application in deciphering HIV-1 transmission is limited [101,102,103]. While these phylogeographic models can reveal and quantify the movement of viruses between locations, they are limited in the in-depth determination of how and where virus transmission has occurred without additional information, e.g., on human movement. Residents in a community may get infected while living or travelling outside their homes, and such external introductions could be further disentangled by combining movement and migration data with virus data. However, foreseeable hurdles include obtaining mobility data from telecommunication companies as well as individual rights protection issues. Third, many phylogenetic studies in sSA have been constrained by low sampling and limited geographic coverage. This limits the extent to which the entirety of HIV-1 transmission dynamics in a country may be characterised. A low sampling density generally results in missing links and smaller clusters of HIV-1 sequences and may therefore limit the reliability of phylogenetic evidence in guiding policy decisions [76]. A potential solution to this problem would be for studies in sSA to aim to increase sampling efforts to achieve larger and proportional sample coverage across all risk groups and geographic locations. Another related challenge is skewed sampling between risk groups and locations resulting in the over representation of some populations and, as a result, a bias in the phylogenetic assessment of transmission dynamics and trait linkage. In the absence of dense sampling, some insights may be accomplished through subsampling available datasets relative to HIV-1 prevalence per risk group or geographic location for proportional representation, albeit with a loss of links due to exclusion of some sequences [8,9,12]. Fourth, a substantial number of published sequences lack information on patient demographics, sampling location, and sampling date, hence limiting their use in phylogeographic studies. In the case of published sequences lacking risk data in sSA, such sequences could be assumed to have been collected from HET individuals (the dominant route of HIV-1 transmission in sSA) [44]. Thereafter, based on phylogenetic clustering, the probable risk group for nodes within a cluster with inadequate annotation may be deduced from association with nodes with a known risk group—as was done to identify potential nondisclosed MSM (self-reported HET men who clustered only with men) in the United Kingdom [104]. With the establishment of the PANGEA consortium (although no data on the contribution of MSM, FSW, or PWID to the epidemic have been reported), a more homogenous and dense sampling from the participating countries may improve and strengthen the limitations of phylodynamic methods [24]. Finally, a potential limitation of our literature search is that it was restricted to studies available only in the PubMed database (https://pubmed.ncbi.nlm.nih.gov/ (accessed on 12 March 2021)). It is therefore possible that some studies were not assessed in our analysis.

## 5. Conclusions

Determining the drivers of the HIV-1 epidemic may be important to guide targeted HIV-1 prevention [29]. Phylogenetic methods could help in characterising such drivers but rely on the availability of large numbers of sequences obtained from well-characterised cohorts. Where these criteria have been achieved (e.g., in European and Northern American settings with dense sampling among infected individuals and patient demographics), phylogenetic studies have provided useful information for HIV-1 prevention [13,16,18,20,21,104,105,106]. Low sampling density is a constant limitation to phylogenetic studies in Africa, and the shortage of HIV-1 sequences from key and vulnerable populations has limited our understanding of the contribution of these populations to the HIV-1 epidemic in sSA. Where data involving populations that are at high risk for HIV-1 infection (such as young girls and fishing communities) are available in sSA, phylogenetic characterisation of sources and directionality of HIV-1 transmission involving these vulnerable populations has been achieved [63,90,93,98,99]. Likewise, if HIV-1 sequences from HIV-1 key populations (i.e., MSM, PWID, and FSW) are made available, phylogenetic studies may guide understanding HIV-1 transmission dynamics and contemporary drivers in these populations. Phylogenetic studies analysing densely sampled and well-characterised HIV-1 key and vulnerable populations sampled in recent years from multiple geographic locations may play a key role in identifying patterns that could be useful in informing HIV-1 prevention strategies in sSA. Overall, although limited, available data from different studies suggest that epidemics among MSM and PWID are more separated and could thus be targeted to reduce population-level incidence. Given that limited HIV-1 sequence data in Africa may continue to present a challenge in the unforeseen future, there is a need to develop statistical and or phylogenetic models that could control for missed sampling.

## Figures and Tables

**Table 1:** Summary of HIV-1 phylogenetic studies in sSA from geographical context.

Country	HIV-1 Subtype	Estimated Date of Introduction	Summary of the Main Findings	PMID ^1^
Central and West African countries				
	F1	1958 (1934–1973)	Spread from DRC, derived from a single founder event. “Pure” F1 variants are most common in Angola.	19386115
Angola	F1	1983 (1978–1989)	The Angolan civil war was associated with a wave of emigration and a phase of negative migratory outflow during 1960–1980.	22484759
	C	1978 (1973–1985) 1979 (1973–1985) 1983 (1977–1990) 1990 (1982–1997) 1994 (1989–1998) 2005 (2002–2008)	HIV-1 subtype C epidemic in Angola originated from multiple independent introductions from Burundi, Zambia, Zimbabwe, and South Africa. The civil war (1974–2002) may have contributed to the emergence of the HIV-1 epidemic in Angola.	22634597
	J, H	Not available	HIV-1 subtypes J and H seem to have been present in Angola since at least 1993.	27098898
	Group M	1978 (1975–1985)	The majority of sequences sampled in 2008–2010 in Luanda clustered together which is consistent with a locally fuelled epidemic.	25479241
Cameroon	CRF02_AG	1973 (1972–1975)	Two distinct lineages of CRF02_AG seem to have ignited in the urban centre of Cameroon. Ethnographic data suggests that well-supported HIV-1 migration was related to chance exportation events rather than by sustained human migratory flows.	21565285
	CRF02_AG	1976 (1966–1984) 1976 (1968–1986) 1979 (1953–1989)	Three monophyletic variants were identified and emerged in the mid-1970’s and spread slowly over 30 years. Continuous exchange of HIV-1 strains between Cameroon and other African countries.	21453131
DRC ^2^	A1, C, D	The 1960s	HIV-1 subtype C origin was estimated to originate in Mbuji-Mayi in the 1950s and subtypes A1, D originated in Kinshasa. The earliest dispersal events of subtype C occurred in a mining region close to Mbuji-Mayi and Lubumbashi. Subtype C spread at least three-fold faster than other subtypes circulating in Central and East Africa.	31809523
DRC ^2^, RC ^3^	Group M	1920 (1909–1930)	Kinshasa estimated to be the origin of the HIV-1 group M pandemic. HIV-1 spread to Brazzaville in the Republic of the Congo, and Lubumbashi and Mbuji-Mayi in the 1930s, which were better connected to Kinshasa, indicating a critical role of mobility networks in the early spread and establishment of the HIV-1 epidemic from the epicentre.	25278604
DRC ^2^, RC ^3^			General Eastward and Southward trends in the spread of HIV-1 from the Kinshasa–Brazzaville and the Pointe-Noire areas to other population centres.	27798403
Guinea-Bissau	CRF02_AG A3	1981 (1974–1986) 1976 (1968–1982) 1980 (1974–1984) 1979 (1972–1984) 1981 (1975–1985) 1979 (1960–1988)	Multiple introductions of CRF02_AG 1976–1981, and a single introduction of sub-subtype A3 in 1979 (median estimates). HIV-1 was introduced into the urban centre (the Capital Bissau) from where it spread to rural areas.	21365013
Nigeria	G CRF02_AG CRF43_02G	1975 (1969–1982) 1963 (1948–1974) 1970 (1960–1980) 1960 (1947–1974) 1971 (1952–1983)	Urban areas (Abuja and Lagos) were the major hubs of HIV-1 transmission in Nigeria. HIV-1 first emerged and expanded within large urban centres before migrating to smaller rural areas.	32103028
East and Southern African countries				
Botswana	C	1996–2002	Presence of multiple phylogenetically distinct HIV-1 subtype C variants (subepidemics) circulating in Mochudi with limited lifespans and temporal dominance. None of the sequences from a rural community of Mochudi clustered with non-Botswana sequences.	26616041 24349005
Ethiopia	C	1965 (1959–1973)	Reconstruction of the epidemic history in Ethiopia revealed that subtype C likely originated from a single lineage in the late 1960s.	20539092
	C	1980	Evidence of clustering between Gondar sequences and sequences from East Africa.	30304061
Kenya	A1	1985–2012	Kilifi sequences clustered closely with sequences from Kenya and other parts of Africa, including West Africa. HIV-1 has been introduced in coastal Kenya multiple times.	32317722
South Africa	C	1960 (1956–1964)	Johannesburg was identified as the hub of HIV-1 dissemination in South Africa. The central region of KwaZulu-Natal was identified as the most likely ancestral location for HIV-1transmission in South Africa for 2 of 14 variants.	26574165
	C	1979–1992	The HIV-1 epidemic in South Africa is suggested to have multiple, parallel subepidemics spreading in the country at the same time.	30804361
	C	1990–2000	Early HIV-1 epidemic dynamics in KwaZulu-Natal were largely driven by external introductions.	30555720
Uganda	A1	1960 (1950–1968)	Ugandan epidemics originated in rural Southwestern Uganda with subsequent spread to other locations without any substantial HIV-1 introductions into this location suggesting that emerging infections from this low-incidence location are mostly from within the region.	25724670
	D	1973 (1970–1977)		33182587
Beyond borders				
West and Central Africa	CRF02_AG	1980 (1978–1981)	CRF02_AG originated from Cameroon from where it spread to other Central and West African countries.	27063411
West and Central Africa	CRF02_AG	1967 (1961–1974) West African	Five different CRF02_AG variants, four of which were restricted to Cameroon and one that grew out into West Africa.	27180893
West and Central Africa	CRF11_cpx	1957 (1950–1966)	Cameroon as the epicentre of dissemination of CRF11_cpx to Central African Republic, Chad, Gabon, and Equatorial Guinea.	27852214
West Africa	CRF06_cpx	1979 (1970–1985)	Burkina Faso was the hub of dissemination of CRF06_cpx to Mali, Nigeria, and the rest of western Central Africa.	23343915
West and Central Africa	G	1974 (1966–1981) 1979 (1973–1984)	Subtype G epidemic clustered into two clusters according to sequence location, i.e., either West or Central Africa. Sequences from West Africa were further subdivided into two large monophyletic clusters that were nested within the Central African variant.	24918930
East Africa	C	1962 (1942–1975)	Subtype C sequences from East Africa (Burundi, Ethiopia, Kenya, Tanzania, and Uganda) formed one large monophyletic cluster separate from sequences from Southern Africa.	22848653 29884822
East Africa	A1 D	1948 (1958–1967)	Both subtype A1 and subtype D were suggested to have spread exponentially during the 1970s.	19644346
East and Southern Africa	C	Not available	The largest number of HIV-1 introductions into South Africa came from Zambia, followed by Botswana, Malawi, and Zimbabwe between 1985 and 2000, a period of mass inward immigration from neighbouring countries into South Africa.	27421210
Zimbabwe	C	1972 (1979–1981)	Multiple cross-border independent introductions of subtype C HIV-1 into Zimbabwe between 1979 and 1981.	19770693

^1^ PMID: PubMed identifier or PubMed unique identifier; ^2^ DRC: The Demographic Republic of Congo; ^3^ RC: The Republic of the Congo.

## Data Availability

No new data were created or analysed in this study. Data sharing does not apply to this article.
